# Developmental Toxicity and Thyroid-Disrupting Effects of Combined Exposure to Pb(II) and ^210^Pb(II) in Zebrafish Embryos

**DOI:** 10.3390/toxics14050372

**Published:** 2026-04-26

**Authors:** Chao Xu, Yuanzhen Li, Lisha Chen, Lujie He, Ruihan Xu, Tianyang Li, Lili Niu, Weiping Liu, Zili Guo, Chenjian Hu

**Affiliations:** 1 Zhejiang Key Laboratory of Low-Carbon Control Technology for Industrial Pollution, Zhejiang University of Technology, Hangzhou 310032, China; chaoxu@zjut.edu.cn (C.X.); 16639420509@163.com (Y.L.); chenlisha0257@163.com (L.C.); xuruihan927@163.com (R.X.); 17857312103@163.com (T.L.); 2 College of Environment, Zhejiang University of Technology, Hangzhou 310032, China; 3 Hangzhou Environmental Protection Science Research and Design Co., Ltd., Hangzhou 310014, China; 19817127550@163.com; 4 Zhejiang Collaborative Innovation Center for Full-Process, Zhejiang Shuren University, Hangzhou 310015, China; wliu@zju.edu.cn (W.L.); guozili@zjsru.edu.cn (Z.G.); 5 Green Governance of Emerging Contaminants, Interdisciplinary Research Academy (IRA), Zhejiang Shuren University, Hangzhou 310015, China; 6 College of Biological and Environmental Engineering, Zhejiang Shuren University, Hangzhou 310015, China; 7 MOE Key Laboratory of Environmental Remediation and Ecosystem Health, Institute of Environmental Health, College of Environmental and Resource Sciences, Zhejiang University, Hangzhou 310058, China; 8 Zhejiang Radiation Environment Monitoring Station, Hangzhou 310012, China

**Keywords:** radioactive metals, combined effects, aquatic toxicity, endocrine disruption, developmental toxicity

## Abstract

The toxicity of radioactive metals arises from both chemical toxicity and radiotoxicity. ^210^Pb(II) is a long-lived radionuclide in the decay chain of natural uranium series ^238^U and exhibits extremely high toxicity. Both ^210^Pb(II) and Pb(II) are widely present in natural water bodies. However, their combined toxicity remains unclear. Based on this, this study used zebrafish embryos as model organisms to investigate developmental toxicity, behavioral toxicity, and thyroid disruption effects, through single and combined exposure to Pb(II) (0, 1, 10, 100 μg/L) and ^210^Pb(II) (0, 100, 1000 Bq/L) for 120 h by comparing the radiotoxicity of ^210^Pb(II) with the chemical toxicity of Pb(II) and further exploring their combined effects. The results showed that following exposure to different environmental concentrations of Pb(II) and environmental activity levels of ^210^Pb(II), exposure to Pb(II) alone caused an increase in the malformation rate of zebrafish embryos, a decrease in locomotor activity, and significant upregulation of thyroid-related genes, including thyroid hormone receptor beta (*TRβ*) and corticotropin-releasing hormone (*CRH*) in the larvae. Exposure to ^210^Pb(II) alone had no significant effects on zebrafish embryos. Notably, compared with the individual exposure groups, the toxic effects in the combined exposure group of Pb(II) and ^210^Pb(II) were further significantly enhanced. Furthermore, correlation analysis indicated a positive correlation between malformations in zebrafish embryos and the expression of key genes in the hypothalamic–pituitary–thyroid (HPT) axis. These findings suggest that under combined exposure, the chemical toxicity of Pb(II) plays a dominant role, while the radioactive component ^210^Pb(II) exerts a synergistic amplification effect. This study provides important scientific evidence for improving the environmental risk assessment of radioactive metals.

## 1. Introduction

Metal ions are widely present in natural water bodies, and their chemical toxicity has been extensively recognized. However, radioactive metals, though also widely present in the natural environment, occur at lower concentrations [1], and their radioactivity is an important toxic characteristic. Through ionizing radiation, radioactive metals induce oxidative stress and DNA double-strand breaks, resulting in radiotoxicity [2,3]. Therefore, chemical toxicity and radiotoxicity may exert combined toxic effects on organisms through synergistic or additive actions. As a long-lived daughter product of the ^238^U decay chain, ^210^Pb(II) exhibits high persistence and bioaccumulation potential, making it a typical environmental risk factor among such radioactive metals [4,5,6]. Its environmental prevalence is increasing with the expansion of nuclear energy and technologies, entering aquatic ecosystems via pathways such as mine-tailings leaching and atmospheric deposition [7,8]. Consequently, ^210^Pb accumulation has been documented in various aquatic organisms, including fish and shellfish, emphasizing its role as a representative radioactive metal contaminant [9].

The chemical toxicity of Pb(II) has been extensively studied. Substantial evidence shows that Pb(II) exposure induces developmental toxicity [10,11], neurotoxicity [12,13], and thyroid endocrine disruption [14] in aquatic vertebrates such as zebrafish. For example, Pb(II) exposure in zebrafish embryos causes severe developmental malformations, behavioral alterations, and the dysregulated expression of related genes [15]. In contrast, the radiotoxicity of ^210^Pb stems from the decay process, which releases high-linear energy transfer (high-LET) α particles. These particles cause localized DNA damage and induce sustained oxidative stress [16]. Notably, even low-dose α-particle irradiation has been shown to trigger significant apoptosis in developing zebrafish embryos, underscoring its potent biological impact [17].

The early developmental stage of vertebrates is a critical period for cell differentiation and organ formation, rendering organisms highly sensitive to environmental pollutants. Studies have shown that exposure to heavy metals such as cadmium (Cd), lead (Pb), and mercury (Hg) can cause malformations in fish embryos and impair neural and cardiac development and behavioral functions, thereby reducing the survival capability of larvae in natural environments [18,19,20]. The underlying toxic mechanisms involve the induction of oxidative stress, DNA damage, and apoptosis [21,22].

The thyroid system plays a central regulatory role in the early development of fish, and its homeostasis depends on the precise regulation of the hypothalamic–pituitary–thyroid (HPT) axis. In this axis, upstream corticotropin-releasing hormone (*CRH*) and thyroid-stimulating hormone (TSH) play key roles in promoting the secretion of thyroid hormones (THs). Additionally, peripheral deiodinases (*Dio1*, *Dio2*) are critically involved in the binding of THs to thyroid hormone receptors (*TRs*). Pb(II) has been demonstrated to disrupt the physiology of thyroid hormones (THs) at multiple levels within this axis, leading to growth and developmental abnormalities and metabolic disorders in organisms [23,24]. For example, in Bufo gargarizans, Pb(II) exposure significantly inhibited the gene expression of thyroid hormone receptor β (*TRβ*) and deiodinases, disrupting THs and interfering with the osteogenic process [25]. Similarly, studies in fish have confirmed that Pb(II) exposure led to thyroid tissue damage, decreased T3/T4 levels, and altered the expression of thyroid-related genes in zebrafish [14,26]. On the other hand, thyroid tissue is highly sensitive to radiation. This characteristic has been widely validated in studies involving internal exposure to radioactive iodine (^131^I) [27] and external gamma irradiation [28]. α-particles released from ^210^Pb decay can disrupt the thyroid homeostasis of rats (Rattus norvegicus), leading to developmental and metabolic dysfunction. The underlying mechanisms include direct damage to thyroid follicular cells, the induction of oxidative stress, and interference with hormone-synthesizing enzymes [29,30,31]. Although the chemical toxicity of Pb(II) and the radiotoxicity of ^210^Pb(II) can each independently induce thyroid disruption, the combined toxic effects of the two on the thyroid and the dominant mechanisms involved remain unclear.

The zebrafish (*Danio rerio*) embryo, owing to its optical transparency, rapid development, and high genetic homology to humans, is an ideal model for assessing the developmental and endocrine-disrupting toxicity of pollutants. In this study, we hypothesize that chemical toxicity and radiotoxicity possess distinct toxic mechanisms of action and may exert combined toxic effects. Therefore, zebrafish embryos were used in this study to investigate the interactive effects of and compare the chemical toxicity of Pb(II), the radiotoxicity of ^210^Pb(II), and their combined toxic effects. By analyzing the development endpoints, locomotor behavior, and transcriptional profiles of HPT axis-related genes, this study aims to provide a scientific basis for improving the environmental risk assessment of radioactive metals.

## 2. Materials and Methods

### 2.1. Chemicals and Reagents

A standard aqueous solution of ^210^Pb (in a PbCl_2_ matrix, with a mass concentration of 2.2824 × 10^−12^ g/g and an activity concentration of 81,272 Bq/L) was provided by the Zhejiang Provincial Radiation Environmental Monitoring Station. PbCl_2_ (analytical grade) was purchased from Shanghai Aladdin Bio-Chem Technology Co., Ltd., Shanghai, China. The ReverTra Ace qPCR RT Kit and SYBR Green Realtime PCR Master Mix were purchased from Toyobo Co., Ltd. (Osaka, Japan). Random primers were purchased from Sangon Biotech (Shanghai) Co., Ltd., Shanghai, China. All other chemicals used in this study were of analytical grade or high-performance liquid chromatography (HPLC) grade.

### 2.2. Zebrafish Maintenance and Embryo Collection

Wild-type AB strain zebrafish (aged 2–3 months) were purchased from the Institute of Hydrobiology, Chinese Academy of Sciences (Wuhan, China). The zebrafish were cultured in a recirculating aquaculture system with dechlorinated tap water. The acclimation conditions were maintained at a temperature of 28 ± 0.5 °C under a 14 h:10 h light–dark photoperiod. They were fed brine shrimp twice daily (morning and evening) for an acclimation period of 1–2 months. Healthy, sexually mature zebrafish were selected and placed in spawning boxes at a female-to-male ratio of 1:2. Spawning was induced the next morning by light stimulation. Embryos were collected within 2 h post-fertilization and rinsed three times with deionized water to remove impurities and dead eggs. They were then disinfected with 1% (*w*/*v*) methylene blue solution (Fujian Weizhenyuan Medical Technology Co., Ltd., Sanming, Fujian, China). After disinfection, the solution was replaced with embryo medium (hardness 100 mg/L CaCO_3_).

### 2.3. Exposure Experiment

The exposure concentrations of Pb(II) in this study were established based on environmentally relevant levels and established water quality criteria. Monitoring data indicate that the concentration of Pb(II) in surface water in a certain region of China ranges from 5 to 32 μg/L (mean: 10 μg/L), with concentrations exceeding 100 μg/L at some sampling sites [32]. Taking into account the chronic exposure criteria proposed by the United States Environmental Protection Agency (USEPA) (0.37–41.0 μg/L) [33], the concentrations of 1 μg/L, 10 μg/L, and 100 μg/L were established in this study, and these concentrations were prepared by the dilution of a 1 mg/L PbCl_2_ stock solution.

To investigate sublethal toxic effects at environmentally relevant levels, the concentrations of ^210^Pb(II) were selected based on available environmental data and regulatory benchmarks. In the absence of specific water quality criteria for ^210^Pb, and given its intrinsic relationship with ^222^Rn (as a significant decay product) in risk assessment, environmental standards for ^222^Rn were referenced. The United Nations Scientific Committee on the Effects of Atomic Radiation (UNSCEAR) reports that up to 10% of global water sources exceeded 100 Bq/L for ^222^Rn [1], while the regulatory limit in Sweden is 1000 Bq/L [34,35]. Preliminary toxicity tests determined the 96 h-LC_50_ of ^210^Pb(II) for zebrafish embryos to be 2528.3 Bq/L (see Supplementary Table S1 for detailed pre-experiment design). Accordingly, nominal activity concentrations of 100 Bq/L and 1000 Bq/L were selected to represent typical environmental levels and an upper regulatory reference limit, respectively, both below the sublethal threshold, and these concentrations were prepared by the dilution of a ^210^Pb(II) standard aqueous solution.

Based on this conversion, the chemical mass concentration of Pb(II) corresponding to the 1000 Bq/L exposure activity of ^210^Pb(II) is 2.8 × 10^−5^ μg/L. This value is not only far below the lowest Pb(II) concentration tested in this study (1 μg/L) but also significantly lower than the concentration range reported to induce early developmental toxicity in zebrafish [36,37,38]. Therefore, under the present experimental conditions, any biological effects observed following ^210^Pb(II) exposure can be attributed primarily to radiotoxicity, with a negligible contribution from its chemical toxicity (see Appendix A for details). All radioactive operations were conducted under compliant radiation protection conditions.

This experiment established a blank control group (embryo medium) and a total of 11 exposure groups: in the single exposure experiments, the concentrations of Pb(II) were 1 μg/L, 10 μg/L, and 100 μg/L; the activity levels of ^210^Pb(II) were 100 Bq/L and 1000 Bq/L. Additionally, six combined exposure groups of Pb(II) + ^210^Pb(II) were set up, namely 1 μg/L + 100 Bq/L, 1 μg/L + 1000 Bq/L, 10 μg/L + 100 Bq/L, 10 μg/L + 1000 Bq/L, 100 μg/L + 100 Bq/L, and 100 μg/L + 1000 Bq/L.

Zebrafish embryos at 4 h post-fertilization (hpf) with normal development were selected. A portion of the embryos were placed in 6-well cell culture plates and exposed until 120 hpf for developmental and behavioral toxicity assessment (15 embryos per well in 10 mL exposure solution). Another portion of the embryos were transferred to beakers and also exposed until 120 hpf for thyroid-related gene transcription level analysis (30 embryos per beaker in 20 mL exposure solution). Exposure was conducted under a 14 h:10 h light/dark cycle at 28 ± 0.5 °C. To maintain experimental stability and consistency, 50% of the exposure solution was renewed daily, and dead embryos were promptly removed. Embryo mortality was determined by coagulation, complete cessation of heartbeat, absence of tail detachment from the body, or arrested development. Each exposure was conducted in triplicate, with the inclusion of a blank control group.

### 2.4. Developmental Toxicity Analysis

Fifteen embryos were used per experimental group (*n* = 3, total 45 embryos). An inverted dissecting microscope (Ti-S, Nikon Corporation, Tokyo, Japan) was used to monitor and record the mortality rate, hatching rate, and malformation rate at 48 h, 72 h, and 96 h.

### 2.5. Locomotor Behavior Analysis

At 120 hpf, eight to ten normally developed zebrafish larvae were randomly selected from each experimental group (*n* = 3). Larvae were individually transferred to a 96-well plate (one larva per well in 100 μL deionized water), with outer wells left empty to eliminate edge effects. Locomotor activity was assessed using a zebrafish behavior analysis system (ZebraLab, ViewPoint Life Sciences, Civrieux, France), following an adapted protocol [39]. After a 20 min light adaptation period, larval motor activity was continuously monitored for 50 min under alternating 10 min light/dark cycles.

### 2.6. Gene Expression Analysis

At 120 hpf, 15 normally developed zebrafish larvae were randomly selected from each experimental group (*n* = 3, total 45 larvae per treatment) for HPT axis-related gene expression analysis. Total RNA was extracted using TRIzol reagent (Takara Biochemicals, Dalian, China) according to the manufacturer’s protocol. RNA concentration and purity were assessed using an ultra-micro-UV spectrophotometer (BioTeke ND5000, Beijing, China), with quality verified by the 260 nm/280 nm absorbance ratio. First-strand cDNA was synthesized using a reverse transcription kit (TOYOBO, Osaka, Japan). Quantitative real-time PCR (RT-qPCR) was performed on a CFX Real-Time PCR System (Bio-Rad, Hercules, CA, USA) using a SYBR Green kit (TOYOBO, Osaka, Japan) in a total volume of 10 μL. The thermal cycling conditions were as follows: pre-denaturation at 95 °C for 1 min, followed by 40 cycles of denaturation at 95 °C for 15 s and annealing/extension at 60 °C for 1 min. β-actin served as internal reference gene, and relative expression levels were calculated using the 2^−ΔΔCT^ method. Primer sequences for the HPT axis-related genes are shown in Table 1.

### 2.7. Statistical Analysis

Statistical analysis was conducted with SPSS 19.0 software (SPSS Inc., Chicago, IL, USA). Data plotting was performed using Origin 9.1 software (OriginLab Corporation, Northampton, MA, USA). All results are expressed as the mean ± standard deviation (mean ± *SD*). Prior to analysis, the homogeneity of variances was examined using Levene’s test. Accordingly, a one-way analysis of variance (ANOVA) followed by Tukey’s multiple comparison test was used to analyze significant differences between the control group and each experimental group, whereas Dunnett’s T3 test was employed for pairwise comparisons among all groups. Statistical significance was set at *p* < 0.05, with significant differences indicated by * in figures and tables.

## 3. Results

### 3.1. Embryonic Development and Behavioral Toxicity

**Figure 1:**
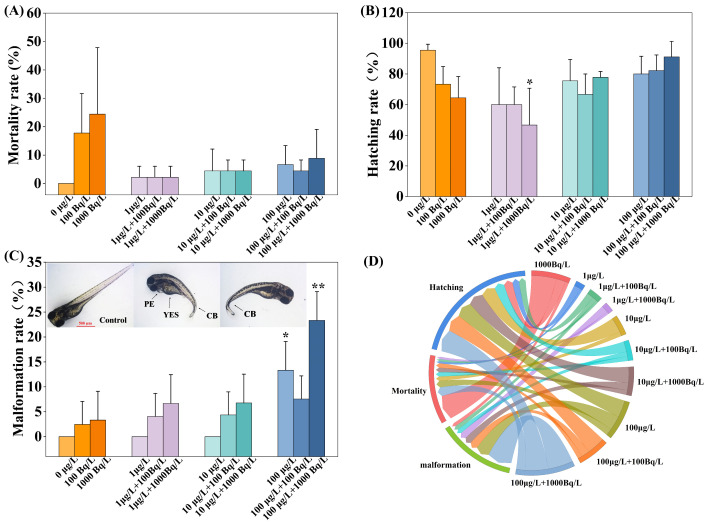
Developmental toxicity in zebrafish embryos following single and combined exposures to Pb(II) and ^210^Pb(II). (**A**) Mortality rate at 48 h post-fertilization (hpf). (**B**) Hatching rate at 72 hpf. (**C**) Malformation rate at 96 hpf, with malformation types including yolk sac edema (YSE), pericardial edema (PE), and curved body (CB). (**D**) Chord diagram illustrating correlations among endpoints. * *p* < 0.05 indicates statistically significant difference; ** *p* < 0.01 indicates highly statistically significant difference.

Following 96 h single and combined exposures to Pb(II) and ^210^Pb(II), the 24 h mortality, 72 h hatching, and 96 h malformation rates of zebrafish embryos were affected to varying extents. Mortality rates are presented in Figure 1A. Relative to the blank control group, the mortality rates in the single ^210^Pb(II) exposure groups (100 Bq/L and 1000 Bq/L) increased to 17.8% and 24.4%, respectively, exhibiting a concentration-dependent trend, though without statistical significance. In the single Pb(II) exposure groups (1, 10, and 100 μg/L), mortality rates increased to 2.2%, 4.4%, and 6.7%, respectively, which likewise did not differ significantly from the control. No significant differences in mortality were observed between the combined exposure groups and the single Pb(II) exposure groups. Hatching rates are presented in Figure 1B. Both single Pb(II) and single ^210^Pb(II) exposure inhibited the hatching rate of zebrafish embryos to varying degrees, although these effects were not statistically significant. However, in the combined exposure group (1 μg/L Pb(II) + 1000 Bq/L ^210^Pb(II)), the hatching rate was significantly reduced to 46.7%. This reduction may be attributed to the combined chemical and radiotoxic effects, suggesting that the synergistic inhibition of hatching by Pb(II) and ^210^Pb(II) may be concentration-dependent. Malformation rates are presented in Figure 1C. Single ^210^Pb(II) exposure did not significantly affect the malformation rate, whereas single exposure to the highest concentration (100 μg/L) significantly increased the malformation rate to 13.3%. In the high-concentration combined exposure group (100 μg/L Pb(II) + 1000 Bq/L ^210^Pb(II)), the malformation rate further increased to 23.3%. The observed malformations included yolk sac edema (YSE), pericardial edema (PE), and curved body (CB), with pericardial edema being the predominant type. This finding suggests that the chemical toxicity of Pb(II) and the radiotoxicity of ^210^Pb(II) may act synergistically, thereby exacerbating developmental malformations in zebrafish. Figure 1D illustrates the effects of the different exposure groups on the three aforementioned biological endpoints, clearly revealing the pronounced impact of the high-concentration combined exposure group on embryonic development.

**Figure 2:**
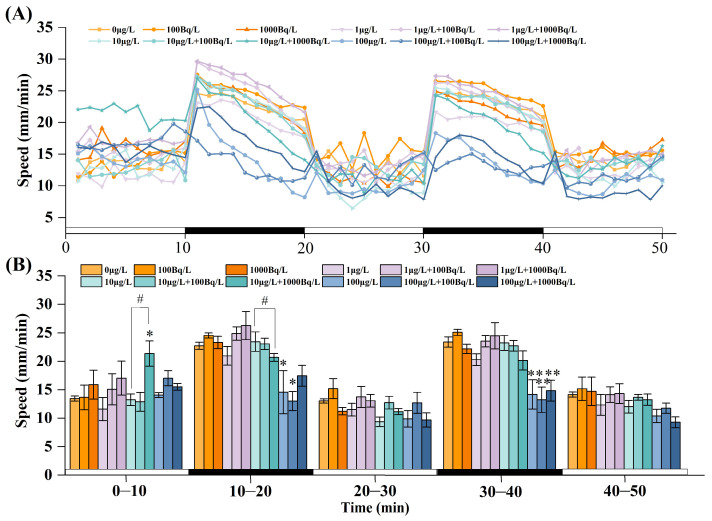
Changes in the average swimming speed of zebrafish larvae during each light/dark cycle following 120 h of single and combined exposure to Pb(II) and ^210^Pb(II): (**A**) The average locomotor speed of larvae per minute. (**B**) The average locomotor speed of larvae per 10 min interval. The white and black bars at the bottom indicate the light and dark periods, respectively. * *p* < 0.05 indicates a statistically significant difference; ** *p* < 0.01 indicates a highly statistically significant difference. # indicates a significant difference between the single Pb(II) exposure group and the corresponding combined exposure group (i.e., with the same Pb(II) concentration) after co-exposure with ^210^Pb(II).

Following 120 h of exposure, the locomotor activity of zebrafish larvae was significantly altered (Figure 2). During the 50 min observation period, the overall activity level of the larvae was significantly higher in the dark phases than in the light phases, exhibiting a typical dark-active, light-inhibited behavioral rhythm (Figure 2A). During the initial light phase (0–10 min), locomotor activity in the combined exposure group (10 μg/L Pb(II) + 1000 Bq/L ^210^Pb(II)) was significantly higher than that in the single exposure group (10 μg/L Pb(II)). In the subsequent dark phase (10–20 min), locomotor activity in the high-concentration single Pb(II) exposure group (100 μg/L) was significantly reduced by 35.7% compared with the control group, whereas the combined exposure group (100 μg/L Pb(II) + 100 Bq/L ^210^Pb(II)) exhibited a reduction of 42.7%. Compared with the single Pb(II) exposure group (10 μg/L), the combined exposure group (10 μg/L Pb(II) + 1000 Bq/L ^210^Pb(II)) exhibited significantly reduced locomotor activity. During the dark phase (30–40 min), locomotor activity in the single Pb(II) exposure group (100 μg/L) was significantly reduced by 39.3% compared with the control group, whereas the combined exposure groups (100 μg/L Pb(II) + 100 Bq/L ^210^Pb(II) and 100 μg/L Pb(II) + 1000 Bq/L ^210^Pb(II)) exhibited reductions of 43.6% and 36.7%, respectively. However, single exposure to ^210^Pb(II) had no significant effect on larval locomotor activity (Figure 2B). These results indicate that single exposure to medium and high concentrations of Pb(II) significantly inhibited locomotor activity in zebrafish larvae and that the addition of ^210^Pb(II) may further exacerbate this inhibitory effect.

### 3.2. Gene Expression Related to the HPT Axis in Larval Fish

**Figure 3:**
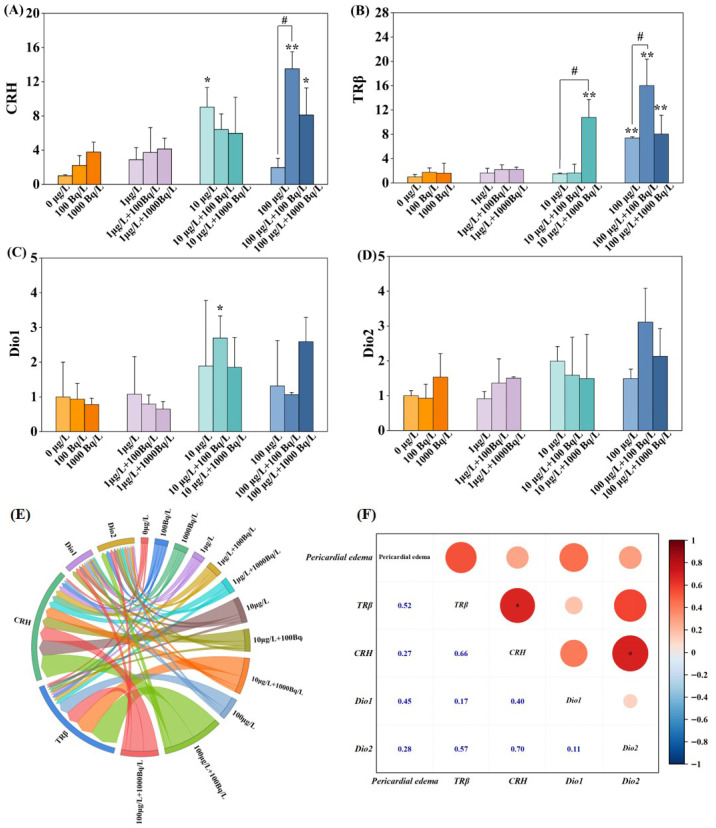
Expression of hypothalamic–pituitary–thyroid (HPT)-related genes in zebrafish larvae following single and combined exposure to Pb(II) and ^210^Pb(II) for 120 h. (**A**) corticotropin-releasing hormone (*CRH*); (**B**) thyroid hormone receptor beta (*TRβ*); (**C**) iodothyronine deiodinase 1 (*Dio1*); (**D**) iodothyronine deiodinase 2 (*Dio2*); (**E**) chord diagram illustrating correlations among HPT axis-related genes; (**F**) correlation analysis between malformation rate of zebrafish embryos and HPT axis-related gene expression. * *p* < 0.05 indicates statistically significant difference; ** *p* < 0.01 indicates highly statistically significant difference. # indicates significant difference between single Pb(II) exposure group and corresponding combined exposure group (i.e., with same Pb(II) concentration) after co-exposure with ^210^Pb(II).

Figure 3 presents the transcriptional levels of hypothalamic–pituitary–thyroid (HPT) axis-related genes in zebrafish larvae following 120 h of single and combined exposure to Pb(II) and ^210^Pb(II). As shown in Figure 3A, corticotropin-releasing hormone (*CRH*) expression was not significantly altered in the single ^210^Pb(II) exposure groups. In the single Pb(II) exposure group (10 μg/L), *CRH* expression was significantly upregulated by 9.0-fold, whereas no significant change was observed in the single Pb(II) exposure group (100 μg/L). However, in the combined exposure groups (100 μg/L Pb(II) + 100 Bq/L ^210^Pb(II) and 100 μg/L Pb(II) + 1000 Bq/L ^210^Pb(II)), *CRH* expression was significantly upregulated by 13.5-fold and 8.1-fold, respectively. Moreover, the combined exposure group (100 μg/L Pb(II) + 100 Bq/L ^210^Pb(II)) exhibited significantly higher expression than the single Pb(II) exposure group (100 μg/L). As shown in Figure 3B, thyroid hormone receptor beta (*TRβ*) expression was not significantly altered in the single ^210^Pb(II) exposure groups. In the single Pb(II) exposure group (100 μg/L), *TRβ* expression was significantly upregulated by 7.4-fold. In the combined exposure groups (10 μg/L Pb(II) + 1000 Bq/L ^210^Pb(II), 100 μg/L Pb(II) + 100 Bq/L ^210^Pb(II), and 100 μg/L Pb(II) + 1000 Bq/L ^210^Pb(II)), *TRβ* expression was significantly upregulated by 10.8-fold, 16.0-fold, and 8.0-fold, respectively. Furthermore, compared with the single Pb(II) exposure group (10 μg/L), the combined exposure group (10 μg/L Pb(II) + 1000 Bq/L ^210^Pb(II)) exhibited significantly upregulated *TRβ* expression. Similarly, compared with the single Pb(II) exposure group (100 μg/L), the combined exposure group (100 μg/L Pb(II) + 100 Bq/L ^210^Pb(II)) exhibited significantly upregulated *TRβ* expression. As shown in Figure 3C, iodothyronine deiodinase 1 (*Dio1*) expression was not significantly altered in any of the single Pb(II) or ^210^Pb(II) exposure groups but was significantly upregulated by 2.7-fold in the combined exposure group (10 μg/L Pb(II) + 100 Bq/L ^210^Pb(II)). As shown in Figure 3D, iodothyronine deiodinase 2 (*Dio2*) expression was not significantly altered in any of the exposure groups. Chord diagram analysis (Figure 3E) revealed that the *CRH* sector exhibited the densest chord distribution, showing significant associations with *TRβ*, *Dio1*, and *Dio2*. The sectors corresponding to high-concentration Pb(II) exposure (100 μg/L) formed strong connections with *CRH* and *TRβ*. The chord distribution was more concentrated in the combined exposure groups, suggesting that ^210^Pb(II) exerts a synergistic effect with Pb(II) at medium and high concentrations, thereby further exacerbating the upregulation of HPT axis-related genes. Correlation analysis (Figure 3F) revealed a positive correlation between malformations in zebrafish embryos and the expression of key HPT axis genes, with a strong correlation for *TRβ*, whereas correlations for *CRH* and *Dio2* were weak. Notably, the addition of ^210^Pb(II) significantly enhanced gene expression levels and the intensity of their interactions, further revealing its synergistic effect with Pb(II) in disrupting the HPT axis. These findings indicate that combined environmental exposure to Pb(II) and ^210^Pb(II) may interfere with the HPT axis through synergistic effects, thereby producing more pronounced developmental toxicity than single exposures.

## 4. Discussion

In this study, we evaluated the toxic effects of single and combined exposure to Pb(II) and ^210^Pb(II) on zebrafish embryos by analyzing developmental toxicity, locomotor behavior, and the transcriptional expression of HPT axis-related genes. The results showed that single exposure to Pb(II) induced developmental toxicity, behavioral toxicity, and thyroid disruption in zebrafish embryos, whereas single exposure to ^210^Pb(II) had no significant effects. However, combined exposure to Pb(II) and ^210^Pb(II) resulted in more pronounced developmental toxicity, behavioral toxicity, and thyroid disruption in zebrafish embryos, which may be attributed to synergistic effects between the two contaminants.

Our study demonstrated that single and combined exposure to Pb(II) and ^210^Pb(II) exerted varying degrees of effects on zebrafish embryonic development, with the hatching rate and malformation rate being more sensitive to combined exposure. Although the inhibitory effects of Pb(II) alone and ^210^Pb(II) alone on the hatching rate did not reach statistical significance, a significant reduction in the hatching rate was observed in the combined exposure group with low-concentration Pb(II) (1 μg/L) and high-radioactivity ^210^Pb(II) (1000 Bq/L). This phenomenon suggests a synergistic interaction between chemical toxicity and radiotoxicity, which may lead to the inhibition of key enzyme activities involved in hatching, damage to the chorion structure, or delayed embryonic development, thereby impeding normal hatching. Pb(II) exposure alone significantly increased malformation rates. This is consistent with previous reports that Pb(II) exposure induces significant developmental toxicity in zebrafish embryos, including reduced hatching, increased mortality, and malformation [40]. Notably, co-exposure further elevated pericardial edema rates, indicating a synergistic effect. The underlying mechanisms may involve complementary pathways. Pb(II) is known to saturate metallothionein (MT) binding capacity, leading to tissue accumulation, the disruption of calcium ion homeostasis, and impaired myocardial contractility, ultimately causing cardiovascular developmental abnormalities [41,42,43]. Concurrently, α-particles released from ^210^Pb(II) decay induce direct DNA damage and apoptosis in cardiomyocytes through ionizing radiation, compromising cardiac contractile function [16]. Thus, we propose that under combined exposure, the toxicities of the two substances are additive and amplify damage, ultimately manifesting as a synergistic enhancement effect on hatching inhibition and increased malformations.

Single Pb(II) exposure significantly inhibited larval swimming speed, consistent with its known effects on motor function. Pb(II) disrupts actin and myosin organization in zebrafish larvae, impairing locomotor ability [44]. Additionally, Pb(II) crosses the blood–brain barrier, accumulates in neural tissues, interferes with neurotransmitter synthesis and synaptic development, and disrupts neural signal transduction, leading to neurobehavioral deficits [44,45,46]. Co-exposure to Pb(II) + ^210^Pb(II) produced more pronounced behavioral toxicity. A paradoxical phenomenon was observed where the swimming speed of the low-concentration combined group was significantly lower than that of the corresponding single Pb(II) group during the dark period, yet it was abnormally elevated during light periods. This observation aligns with previous findings that Pb(II) exposure interferes with N-methyl-D-aspartate receptor (NMDAR)-dependent brain-derived neurotrophic factor (BDNF) signaling, causing abnormal neurobehavior characterized by hypoactivity in dark periods and hyperactivity in light periods [13]. As a radiation source, α-particles from ^210^Pb decay generate reactive oxygen species (ROS) through water radiolysis [2,3], inducing strong oxidative stress. Combined with DNA damage and persistent neuroinflammation, this synergistically exacerbates central nervous system damage, ultimately dysregulating motor control [47,48,49]. We speculate that the significant behavioral inhibition observed during dark periods under co-exposure results from synergistic chemical and radiotoxic effects on neural circuitry. The transient hyperactivity in low-concentration co-exposure groups during light periods may reflect a compensatory stress response to combined low-level stressors, though the underlying mechanisms warrant further investigation.

In zebrafish, the HPT axis is a core pathway regulating the synthesis, secretion, transport, and metabolism of THs, and the orderly transcription of its related genes is essential for maintaining nervous system development and locomotor behavior [50]. Additionally, HPT axis homeostasis plays a critical role in cardiac development [51], and aberrant perturbations in its expression profile are closely associated with developmental malformations such as pericardial edema [52]. However, this finely regulated signaling pathway is highly sensitive to environmental pollutants, and disruptions in gene expression often precede changes in hormone levels, serving as early indicators of toxic effects [52,53]. In this study, a correlation analysis of the transcriptional expression patterns of key HPT axis-related genes revealed that exposure to Pb(II) and ^210^Pb(II) significantly disturbed the regulatory pathways of the HPT axis, particularly the expression changes in key genes such as *CRH* and *TRβ*. This disruption exhibited a synergistic enhancement effect between ^210^Pb(II) and Pb(II) and showed a positive correlation with the occurrence of malformations in zebrafish embryos, providing mechanistic support for the observed developmental toxicity and behavioral abnormalities. Existing studies further indicate that HPT axis dysfunction is an important mechanism driving developmental toxicity and behavioral abnormalities in organisms. For example, uranium (U) exposure interferes with thyroid function by disrupting key HPT axis gene expression, resulting in delayed hatching, malformations, and abnormal swimming behavior in zebrafish embryos [54]. Similarly, ^137^Cs radiation-induced oxidative stress and DNA damage indirectly inhibit thyroid hormone receptor signaling, leading to cognitive impairment in fetal rats [55].

In our study, single exposure to Pb(II) significantly upregulated *CRH* and *TRβ* expression, while *Dio1* and *Dio2* showed no significant changes. As a key stimulator of TSH secretion, *CRH* may be upregulated as a negative feedback response to decreased T4 levels. This aligns with findings that heavy metals such as mercury (Hg) and uranium (U) induce *CRH* upregulation in zebrafish alongside impaired thyroid function, suggesting an adaptive response to maintain thyroid homeostasis [39,56]. *TRβ*, a thyroid hormone receptor that binds T3 to mediate gene expression, shows upregulated transcription as a compensatory response to reduced TH levels [57]. Similar *TRβ* upregulation has been observed in zebrafish exposed to cadmium (Cd) and copper (Cu) [53,58].

In contrast to Pb(II), single exposure to ^210^Pb(II) showed no significant effects on developmental toxicity, behavioral toxicity, or thyroid disruption. Previous studies report conflicting findings: some show that low-dose α-particle irradiation induces apoptosis in dechorionated zebrafish embryos [17], while other research shows no significant apoptosis in 25 hpf embryos [59]. This may explain why ^210^Pb internal irradiation causes early embryonic apoptosis and increased mortality, yet after a 120 h adaptive period, surviving larvae exhibit no significant behavioral or HPT axis effects.

Notably, co-exposure further significantly upregulated *CRH* and *TRβ* expression compared with Pb(II) alone, indicating that ^210^Pb(II) radiotoxicity acts additively or synergistically with Pb(II) chemical toxicity to enhance thyroid disruption. Although the thyroid is not a primary accumulation site for ^210^Pb, α-particles emitted during its decay can induce DNA double-strand breaks in thyroid follicular epithelial cells, triggering cell death and structural damage. This may inhibit key synthesis enzymes such as thyroglobulin (Tg) and thyroid peroxidase (TPO), thereby impairing thyroid hormone (TH) synthesis [60,61]. This chemical–radiotoxicity synergy is supported by studies on other radionuclides. For example, at equal chemical concentrations (100 μg/L), highly radioactive ^233^U induces more pronounced oxidative stress in zebrafish livers than depleted uranium (DU) [62]. However, such interactions exhibit pronounced dose dependency. When zebrafish embryos were exposed to 0.44 mGy α-particles + 10 µg/L DU versus 4.4 mGy α-particles + 10 µg/L DU, the effects were antagonistic, whereas exposure to 4.4 mGy α-particles + 100 µg/L DU exhibited synergy [29]. It is worth noting that in this study, single exposure to ^210^Pb(II) exhibited no significant radiotoxicity, whereas toxicity was significantly enhanced when combined with Pb(II). This may be attributed to the chemical toxicity of Pb(II) lowering the defense threshold, thereby amplifying radioactive damage. Specifically, Pb(II) can significantly weaken fish immune defense through various pathways, including oxidative stress [63,64], immune cell damage [64,65], and a reduction in innate and adaptive immune functions [66,67]. This may serve as an important prerequisite for the amplification of ^210^Pb(II) toxicity under combined exposure conditions.

## 5. Conclusions

In this study, zebrafish embryos were used as model organisms to investigate the toxic effects of single and combined exposure to Pb(II) and ^210^Pb(II). The results showed that within the experimental concentration range, single exposure to Pb(II) caused a significant increase in the malformation rate, a decrease in locomotor activity, and significant upregulation of key genes in the HPT axis, such as *CRH* and *TRβ*. Although single exposure to ^210^Pb(II) did not induce significant toxicity, when combined with Pb(II), it significantly enhanced the aforementioned toxic effects. Correlation analysis indicated that the disruption of key HPT axis genes, including *CRH* and *TRβ*, by combined exposure to Pb(II) and ^210^Pb(II) was positively correlated with the malformation rate in zebrafish embryos. Overall, in the combined toxicity of Pb(II) and ^210^Pb(II), chemical toxicity plays a dominant role, while radiotoxicity enhances the disruptive effects of the combined exposure system on early development, behavior, and thyroid function. Therefore, this study provides new scientific evidence for better understanding the environmental risks of radioactive metals. Furthermore, it should be noted that the developmental abnormalities and behavioral disorders observed in zebrafish embryos are subject to synergistic regulation by multiple pathways and targets. This study only identifies the disruption of the HPT axis as one potential mechanism underlying the combined developmental toxicity of the two agents. However, inferences regarding thyroid-disrupting effects based solely on transcriptional changes require further corroboration through quantitative measurements of thyroid hormones (T3/T4) and the validation of downstream functional protein expression.

## Figures and Tables

**Table 1:** Primer sequences for HPT axis-related genes used in real-time qPCR.

Gene Name	Sequence of the Primer (5′–3′)	Accession Number
*β-catin*	Forward: ATGGATGAGGAAATCGCTGCC	AF057040
	Reverse: CTCCCTGATGTCTGGGTCGTC	
*Dio1*	Forward: GTTCAAACAGCTTGTCAAGGACT	BC076008
	Reverse: AGCAAGCCTCTCCTCCAAGTT	
*Dio2*	Forward: GCATAGGCAGTCGCTCATTT	NM_212789
	Reverse: TGTGGTCTCTCATCCAACCA	
*TRβ*	Forward: TGGGAGATGATACGGGTTGT	NM_131340
	Reverse: ATAGGTGCCGATCCAATGTC	
*CRH*	Forward: TTCGGGAAGTAACCACAAGC	NM_001007379
	Reverse: CTGCACTCTATTCGCCTTCC	

## Data Availability

The data that support the findings of this study are available upon reasonable request from the corresponding author.

## Supplementary Materials

The following supporting information can be downloaded at https://www.mdpi.com/article/10.3390/toxics14050372/s1, Table S1. Experimental design of the pre-experiment for 96 h-LC_50_ determination of ^210^Pb(II) in zebrafish embryos.

## Appendix A

Equation (A1)

The stock solution of ^210^Pb (as PbCl_2_ in aqueous matrix) has a certified activity concentration of A_0_ = 81272 Bq/L and a Pb mass fraction of w_0_ = 2.2824 × 10^−12^ g/g.

(A1) $$C_{0}=(A_{0})/(w_{0})$$

Conversely, a given Pb mass concentration C corresponds to an activity concentration A, Equation (A2)

(A2) $$C=C_{0}\times A/(A_{0})$$
