# Supplementary material for: Developmental Toxicity and Thyroid-Disrupting Effects of Combined Exposure to Pb(II) and 210Pb(II) in Zebrafish Embryos
Source: Toxics. 2026 Apr 26;14(5):372. doi: 10.3390/toxics14050372 (PMC13210810; doi:10.3390/toxics14050372)
Supplement: Supplementary file 1 [file toxics-14-00372-s001.zip › toxics-4247291-supplementary.pdf]

Supplementary Materials

Developmental Toxicity and Thyroid-Disrupting Effects of Combined Exposure to Pb(II) and ^{210}Pb(II) in Zebrafish Embryos

Chao Xu [1,2], Yuanzhen Li [1,2], Lisha Chen [1,2], Lujie He [3], Ruihan Xu [1,2], Tianyang Li [1,2],

Lili Niu [4,5,6,*], Weiping Liu [4,5,6,7], Zili Guo [4,5,6] and Chenjian Hu [8,*]

1 Zhejiang Key Laboratory of Low-Carbon Control Technology for Industrial Pollution, Zhejiang University of Technology, Hangzhou 310032, China; chaoxu@zjut.edu.cn (C.X.); 16639420509@163.com (Y.L.); chenlisha0257@163.com (L.C.); xuruihan927@163.com (R.X.); 17857312103@163.com (T.L.)
2 College of Environment, Zhejiang University of Technology, Hangzhou 310032, China
3 Hangzhou Environmental Protection Science Research and Design Co., Ltd., Hangzhou 310014, China; 19817127550@163.com
4 Zhejiang Collaborative Innovation Center for Full-Process, Zhejiang Shuren University, Hangzhou 310015, China; wliu@zju.edu.cn (W.L.); guozili@zjsru.edu.cn (Z.G.)
5 Green Governance of Emerging Contaminants, Interdisciplinary Research Academy (IRA), Zhejiang Shuren University, Hangzhou 310015, China
6 College of Biological and Environmental Engineering, Zhejiang Shuren University, Hangzhou 310015, China
7 MOE Key Laboratory of Environmental Remediation and Ecosystem Health, Institute of Environmental Health, College of Environmental and Resource Sciences, Zhejiang University, Hangzhou 310058, China
8 Zhejiang Radiation Environment Monitoring Station, Hangzhou 310012, China
* Correspondence: niulili@zjsru.edu.cn (L.N.); cjhu228@sina.com (C.H.)

No. of pages: 5

No. of tables: 1

Pre-Experiment Protocol for 96 h-LC_{50} Determination of $^{210}Pb(II)$ in Zebrafish Embryos

1. Experimental Objective

To determine the 96-hour median lethal concentration (96 h-LC_{50}) of $^{210}Pb(II)$ in zebrafish embryos through a gradient exposure experiment, thereby providing a basis for selecting sublethal and environmentally relevant exposure concentrations for the formal experiment.

2. Experimental Materials and Instruments

Test organism: AB strain wild-type zebrafish embryos at 4 hours post-fertilization (hpf), normally developed, without malformation or mortality

Test substance: $^{210}Pb(II)$ standard aqueous solution (certified reference material, activity concentration of 81272 Bq/L, $PbCl_2$ aqueous matrix)

Culture medium: Zebrafish embryo culture medium (prepared as described in Section 2.2 of the main text; total hardness 100 mg/L $CaCO_3$)

Culture conditions: 28 ± 0.5 °C, 14 h:10 h light/dark cycle

Instruments: Stereomicroscope, constant temperature incubator, 6-well cell culture plates, sterile pipettes, radiation dosimeter

3. Experimental Design

Exposure groups: Five $^{210}Pb(II)$ activity gradient groups were established, covering a range from sublethal to lethal concentrations. A blank control group (embryo culture medium only) was also included. The specific groups were as follows:

Table S1. Experimental design of the pre-experiment for 96 h-LC_{50} determination of $^{210}Pb(II)$ in zebrafish embryos

Group	Concentration (Bq/L)
Blank control	0
Gradient 1	250
Gradient 2	500
Gradient 3	1,000
Gradient 4	2,000
Gradient 5	4,000

Biological replicates and sample size

- Three biological replicates (independent culture wells) per group
- 10 mL of the corresponding exposure solution was added to each well, containing 20 healthy zebrafish embryos at 4 hpf
- Total number of embryos per treatment group: 60

Exposure procedure

- Exposure start time: 4 hpf (consistent with the formal experiment)
- Exposure duration: Continuous exposure for 96 hours
- Solution renewal: 50% of the exposure solution was renewed every 24 hours to maintain stable exposure concentrations
- Radiation safety: All exposure solution preparation and renewal operations were conducted in a radiation protection laboratory. Operators wore personal protective equipment and strictly adhered to national radiation safety regulations.

4. Observation Endpoints and Assessment Criteria

Observation time points: 24, 48, 72, and 96 hpf (observations were made at fixed times daily, and data were recorded)

Mortality endpoint: Embryos were considered dead if they exhibited complete coagulation, complete cessation of heartbeat, and absence of spontaneous somite movement. Dead embryos were promptly removed.

Recorded parameters: Number of dead embryos per well at each time point; cumulative mortality rate (calculated as cumulative number of dead embryos / total number of embryos × 100%).

5. Data Processing and Results

Data were analyzed using SPSS 19.0 (IBM Corp., Armonk, NY, USA). Nonlinear dose-response fitting was applied to the cumulative mortality data at 96 hpf, and a concentration-response curve was generated. The calculated 96 h-LC_{50} of $^{210}Pb(II)$ in zebrafish embryos was **2528.3 Bq/L**.

Based on this result, 100 Bq/L and 1000 Bq/L were selected as sublethal exposure concentrations for the formal experiment (both far below the LC_{50} and within environmentally relevant levels). These concentrations were used to investigate the developmental toxicity, behavioral toxicity, and thyroid-disrupting effects of $^{210}Pb(II)$ at low doses, while avoiding high lethality that could mask sublethal toxic phenotypes.

6. Ethical and Radiation Safety Statement

Animal ethics: This experiment strictly adhered to the guidelines of the Institutional Animal Care and Use Committee. Unnecessary harm was minimized, and surviving embryos were humanely disposed of after the experiment.

Radiation safety: ^{210}Pb used in this experiment is a radioactive nuclide. All exposure solutions, experimental waste liquids, and contaminated consumables were collected in accordance with the *Regulations on the Safety and Protection of Radioisotopes and Radiation Devices* and were transferred to a qualified professional unit for compliant disposal. All experimental procedures complied with radiation safety management requirements, and there was no risk of radiation leakage.
